# Long-term use of implanted peroneal functional electrical stimulation for stroke-affected gait: the effects on muscle and motor nerve

**DOI:** 10.1186/s12984-019-0556-2

**Published:** 2019-07-10

**Authors:** Frank Berenpas, Vivian Weerdesteyn, Alexander C. Geurts, Nens van Alfen

**Affiliations:** 1 Department of Rehabilitation, Donders Institute for Brain, Cognition and Behaviour, Radboud University Medical Center, P.O. Box 9101, 6500 HB Nijmegen, The Netherlands; 2 Department of Neurology and Clinical Neurophysiology, Donders Institute for Brain, Cognition and Behaviour, Radboud University Medical Center, P.O. Box 9101, 6500 HB Nijmegen, The Netherlands

**Keywords:** Stroke, Foot drop, Implanted FES, Ultrasound, TMS, Transsynaptic degeneration

## Abstract

**Background:**

Peripheral changes to muscle and motor nerves occur following stroke, which may further impair functional capacity. We investigated whether a year-long use of an implanted peroneal FES system reverses stroke-related changes in muscles and motor nerves in people with foot drop in the chronic phase after supratentorial stroke.

**Methods:**

Thirteen persons with a chronic stroke (mean age 56.1 years, median Fugl-Meyer Assessment leg score 71%) were included and received an implanted peroneal FES system (ActiGait®). Quantitative muscle ultrasound (QMUS) images were obtained bilaterally from three leg muscles (i.e. tibialis anterior, rectus femoris, gastrocnemius). Echogenicity (muscle ultrasound gray value) and muscle thickness were assessed over a one-year follow-up and compared to age-, sex-, height- and weight-corrected reference values. Compound motor action potentials (CMAPs) and motor evoked potentials (MEPs) were obtained from the tibialis anterior muscle. Generalized estimated equation modeling was used to assess changes in QMUS, CMAPs and MEPs outcomes over the follow-up period.

**Results:**

Echogenicity of the tibialis anterior decreased significantly during the follow-up on the paretic side. Z-scores changed from 0.88 at baseline to − 0.15 after 52 weeks. This was accompanied by a significant increase in muscle thickness on the paretic side, where z-scores changed from − 0.32 at baseline to 0.48 after 52 weeks. Echogenicity of the rectus femoris normalized on both the paretic and non-paretic side (z-scores changed from − 1.09 and − 1.51 to 0.14 and − 0.49, respectively). Amplitudes of CMAP and MEP (normalized to CMAP) were reduced during follow-up, particularly on the paretic side (ΔCMAP = 20% and ΔMEP = 14%).

**Conclusions:**

We show that the structural changes to muscles following stroke are reversible with FES and that these changes might not be limited to electrically stimulated muscles. No evidence for improvement of the motor nerves was found.

**Electronic supplementary material:**

The online version of this article (10.1186/s12984-019-0556-2) contains supplementary material, which is available to authorized users.

## Introduction

Stroke is typically defined as a lesion of the upper motor neuron (UMN). However, it is known that secondary to this UMN lesion peripheral changes occur after a stroke [1, 2]. Following the loss of central activation, lower motor neurons (LMN) may become functionally depressed or may even undergo ‘transsynaptic degeneration’ leading to denervation of muscle fibers [3–5]. Since denervation of muscle fibers induces muscle atrophy and infiltration of fibrous tissue and fat, this process of denervation after stroke also has an effect on the structure of skeletal muscles. Indeed, muscle atrophy and infiltration of fibrous tissue and fat are often reported in paretic muscles after stroke [6–8]. Recently we have shown that structural changes to muscles after stroke are not restricted to the paretic side alone and that the changes in muscle structure cannot be explained solely by disuse [9]. These changes to skeletal muscle structure are believed to further impair functional capacity of people with stroke [8]. Therefore, interventions preventing or mitigating this undesirable involvement of the peripheral nerve system and muscles after stroke are needed.

Functional electrical stimulation (FES) of paretic muscles is a commonly applied method to compensate for severe muscle weakness after stroke. One of the most widely used applications of FES in people with stroke is the activation of the peroneal nerve to reduce foot drop [10]. With peroneal FES, paretic dorsiflexor muscles are electrically activated during the swing phase and early stance of the gait cycle, resulting in an ‘active’ foot elevation [11]. It has been theorized that such electrically induced contractions combined with voluntary contractions can strengthen spinal synapses and induce cortical changes [12]. Indeed, increased excitability, metabolism and reorganization of the motor cortex have been reported after prolonged peroneal FES use in people with a neurological disease, including people with stroke [13–16]. These plastic changes after long-term FES use indicate central motor recovery, which raises the question whether stroke-related changes to skeletal muscles and lower motor neurons can also be reversed with prolonged FES use.

**Fig. 1:**
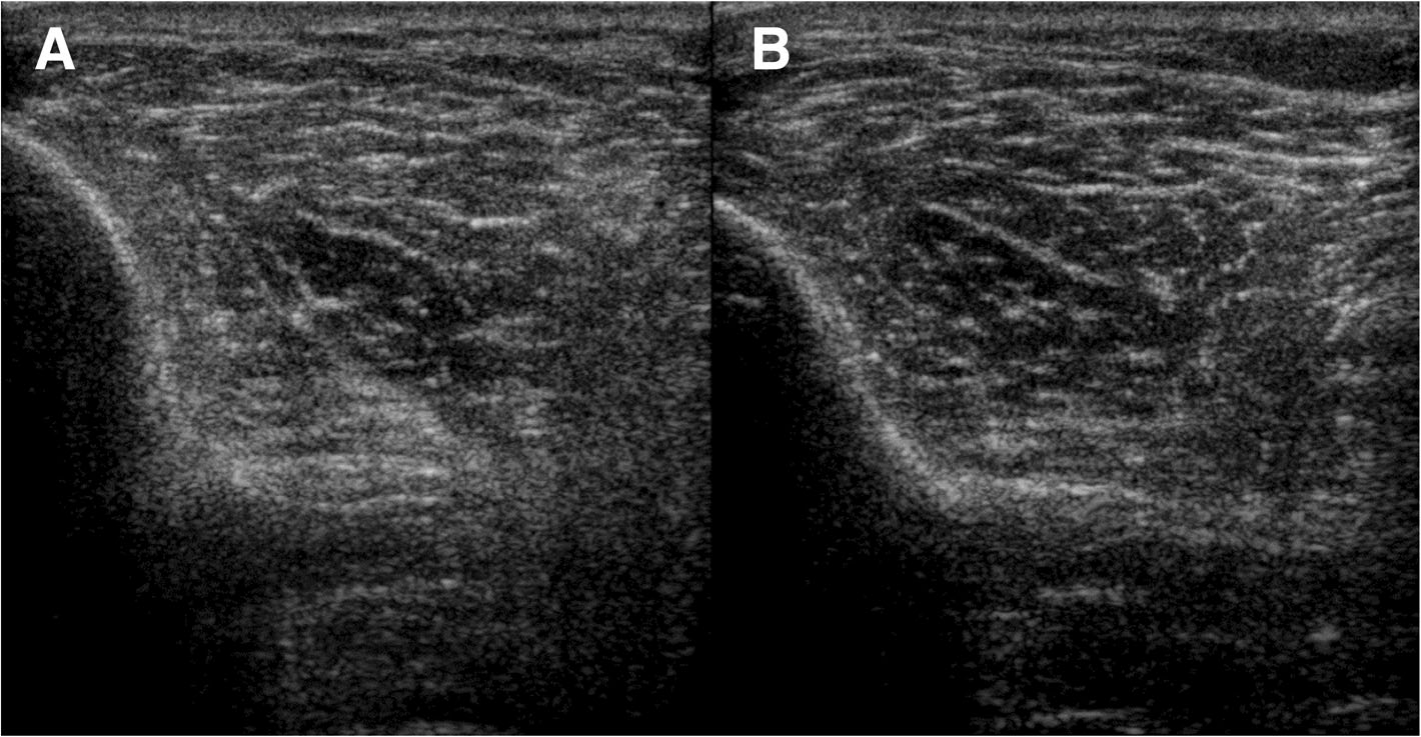
Example of paretic side (**a**) and non-paretic side (**b**) tibialis anterior. The paretic tibialis anterior muscle appears whiter on the screen (i.e. a higher echogenicity), indicating infiltration of intramuscular fat and fibrous tissue. Echogenicity z-scores were z = 1.45 and z = 0.29 for the paretic and non-paretic side, respectively

In this study we aimed to investigate whether a year-long use of an implanted peroneal FES system (ActiGait®, Neurodan, Denmark, Otto Bock Group, 2006) reverses stroke-related changes in skeletal muscles and their motor innervation in people with persistent foot drop in the chronic phase after a supratentorial stroke. Bilateral leg muscle thickness and echogenicity were monitored over time using quantitative muscle ultrasound (QMUS), which is valid method for objective assessment of muscle architecture [17–19] and has been used in various patient populations with neuromuscular and central nervous system disorders [9, 20, 21]. Muscle thickness provides an indication of the presence of muscle atrophy (or hypertrophy), whereas echogenicity (i.e. how white or black the image looks on the screen) is a measure of how (ab-) normal the tissue architecture is. Infiltration of muscle fibers with fat and fibrous tissue following denervation increases muscle echogenicity, making the muscle appear more white on the screen (see Fig. 1 for an example). Furthermore, whether long-term FES stimulation of ankle dorsiflexor muscles changes functioning of the lower motor neurons and alters corticospinal integrity was tested by obtaining compound motor action potentials (CMAPs) and motor evoked potentials (MEPs) from the primary ankle dorsiflexor muscle (i.e. tibialis anterior muscle).

We hypothesized that, following a year-long period of implanted peroneal FES use, muscle architecture would be restored in the electrically stimulated ankle dorsiflexor muscles, but not in other muscles on the paretic or non-paretic side. In addition, as long-term use of FES has been found to induce cortical changes, we hypothesized that MEPs would be increased after a year of FES use. The results of this study will lead to a better insight into the reversibility of architectural changes of muscles and motor nerves after stroke using long-term FES.

## Methods

### Participants

All participants were recruited from the outpatient clinic of the department of Rehabilitation at the Radboud university medical center in Nijmegen and were enrolled in a study evaluating the effects of implanted functional electrical stimulation (FES) on ambulation [22]. Inclusion criteria were: (1) having sustained a supratentorial stroke, i.e. a lesion in one of the cerebral hemispheres, at least 6 months before inclusion, (2) paresis including unilateral foot dorsiflexion weakness (Medical Research Council scale < 5), (3) the ability to walk at least 10 m without a walking aid (except for the use of an ankle-foot orthosis which was allowed) and [4] a positive response to surface-based peroneal nerve stimulation (NESS L300, Bioness inc, Valencia, California) defined as the ability to make initial heel contact during gait with stimulation. Subjects were excluded if: (1) they had a history of (poly-) neuropathy or (poly-) radiculopathy, or (2) were morbidly obese (body mass index > 40), as this can preclude reliable QMUS measurements by attenuation of the ultrasound beam, or (3) reported contraindications for transcranial magnetic stimulation (TMS) (e.g. active epilepsy or the presence of an implanted pacemaker, neurostimulator or cochlear implant). All patients gave their informed consent. The study was approved by the local medical ethics committee and conducted in accordance with the World Medical Association Declaration of Helsinki [23].

#### Demographic and clinical characteristics

Age, sex, time since stroke, type of stroke, body mass index, leg motor strength (Motricity Index [24]) and leg motor selectivity (Fugl-Meyer Assessment [25]) were obtained by the same rehabilitation physician (ACG) at inclusion. In addition, preferred walking speed [26] at baseline and after 52 weeks of implanted FES use was assessed on a 10-m walkway using a stopwatch. Physical activity at baseline and after 52 weeks of FES use was registered by means of a Digi-Walker SW-650 pedometer (Yamax Corporation, Tokyo, Japan) counting the amount of daily steps averaged over a period of 7 days.

### ActiGait® implanted FES system

**Fig. 2:**
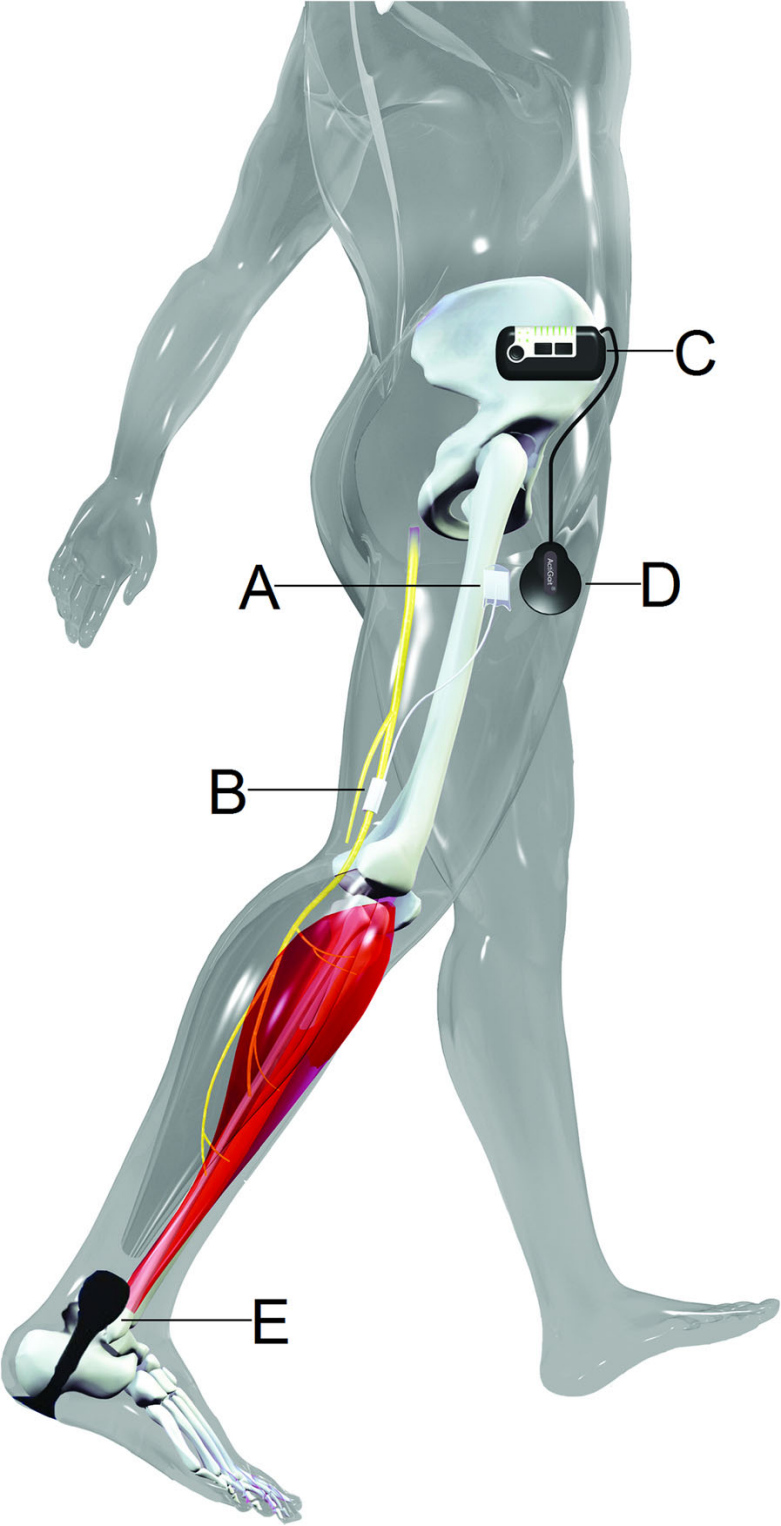
The ActiGait® system. The implanted part of the ActiGait® consists of a stimulator body (**a**) and an electrode cuff (**b**), the latter placed around the common peroneal nerve. The implant is controlled by a control unit (**c**), which activates the implant through an antenna placed on the thigh (**d**). The timing of activation is determined by a heel switch (**e**), which communicates wirelessly with the control unit

The ActiGait® system is an implantable 4-channel peroneal nerve stimulator (Fig. 2). The implant consists of an electrode cuff at the distal end and a stimulator body at the proximal end connected by a lead wire. The electrode cuff has 4 separate electrodes, which are selectively controlled by the stimulator body, allowing differential activation of nerve fibers to the tibialis anterior, peroneus longus/brevis, and toe extensor muscles. The system is operated through external parts: a heel switch (placed under the heel and attached to the shoe or a special sock) and a control unit, worn at the pelvis, which is connected to an antenna on the skin directly over the stimulator body. The control unit enables users to switch the stimulation on and off and to make adjustments in stimulation intensity. The heel switch wirelessly communicates with the control unit to provide information for onset and offset of stimulation.

ActiGait® implantation was performed by a trained neurosurgeon at the Radboud university medical center in Nijmegen. The implant was activated 3 weeks after surgery. Use of the Acitgait® system was then built up gradually in 3 weeks from 15 to 60 min per day in the first week up to minimally 6 h per day in the third week. The procedure of ActiGait® implantation and system activation has previously been described in detail [27–29] and can be found in the Additional file 1 of this report.

### Study design

A within-subjects repeated-measures design was used for the follow-up of 1 year after implantation. QMUS measurements were performed at inclusion (T0) as well as 26 weeks (T3) and 52 weeks (T4) after activation of the ActiGait® system. Neurophysiologic assessments were performed at inclusion (T0) and 2 weeks (T1), 8 weeks (T2), 26 weeks (T3) and 52 weeks (T4) after activation of the ActiGait® system. The effects of long-term implanted FES use on walking capacity have been published elsewhere [22, 29].

### Measurements

#### Quantitative muscle ultrasound

We used QMUS, a method with high interrater reliability [30–32], to assess the primary ankle dorsiflexor muscle (i.e., the tibialis anterior muscle), its antagonist (i.e., the medial head of the gastrocnemius muscle) and the rectus femoris muscle, a biarticular upper leg muscle, all being key muscles for locomotion. Muscle ultrasound images of the tibialis anterior, medial head of the gastrocnemius, and rectus femoris muscles were obtained bilaterally with a Philips IU 22 ultrasound machine (Philips Healthcare Systems, Best, The Netherlands) and a broadband linear 5–17 MHz transducer. A dedicated musculoskeletal preset was used for all scanning, with fixed system parameters set to: gain 70 dB, compression 55, time gain in neutral position, and focal range of 1.0 to 2.5 cm depth. To ensure reproducibility, automatic image optimizing software was turned off as much as possible. System settings remained unchanged throughout all measurements [33]. Muscle ultrasound images were taken at predefined sites corresponding to the maximum muscle thickness of each muscle, following the description of Scholten et al. [34]. At each assessment, three separate muscle ultrasound images were taken from each muscle, all the while ensuring there was no pressure on the skin or the muscle and with the participant in a relaxed, supine position. The digital images were stored as DICOM files for offline analysis. In each image a region of interest (ROI) was selected. Using an image histogram analysis tool, the mean gray value and thickness over the ROIs of separate muscles were calculated with the help of a custom software program (“QUMIA”, [9, 19, 35–37]). This software then compared the muscle thickness and gray values to age-, sex-, height- and weight-corrected reference values [38, 39]. The difference found was expressed as a z-score, i.e. the number of standard deviations (SD) from the mean. Positive z-scores for echogenicity (i.e. muscles looking relatively white on the screen) were considered indicative of poorer muscle architecture with more infiltration of fibrous tissue and fat. Negative z-scores for muscle thickness indicated loss of muscle mass.

#### Electrophysiologic assessments

Surface EMG signals were recorded from the tibialis anterior muscle on both the paretic and non-paretic side. Adhesive electrodes (1 cm diameter) were placed on the muscle belly and muscle tendon of the tibialis anterior muscle. Ground electrodes were placed between the stimulus and recording site. EMG recordings were performed using a multichannel biomedical amplifier (Neurotop MME 3132, Nihon Kohden, Tokyo, Japan).

#### Peripheral motor nerve stimulation

Peripheral motor nerve function was evaluated by measuring the CMAPs from the tibialis anterior muscle. Assessment of CMAPs from the tibialis anterior muscle has been shown to have good test-retest reliability [40]. CMAPs were obtained, for the paretic and non-paretic side separately, by stimulation of the common peroneal nerve, dorsal to the fibular head. Stimulation intensity was increased gradually until an increase in intensity did not further increase the motor-wave (M-wave) amplitude (i.e. supramaximal stimulation). The results of five consecutive stimulations were then recorded and used for analysis. After rectification of the raw signal, the maximum peak-to-peak amplitude and the largest area under the curve (AUC) of these five contractions were extracted offline, using a custom Matlab script (Matlab 20141b, The Mathworks Inc. Natick, Massachusetts).

#### Transcranial magnetic stimulation

For assessment of the corticospinal connections to the tibialis anterior, MEPs were obtained using TMS. The motor cortex was stimulated three times at the vertex with a double-cone coil at maximal stimulator output using a transcranial magnetic stimulator (Magstim 200, Magstim, Whitland, UK). The MEPs were recorded when participants performed a slight voluntary ankle dorsiflexion to enlarge the MEP response. From the three stimuli obtained in each assessment, participants’ maximum peak-to-peak amplitude as well as the largest AUC after rectification of the raw signal were extracted offline using a custom Matlab script (Matlab 2041b, The Mathworks Inc. Natick, Massachusetts). Since MEP output is limited by functioning of the lower motor neuron, MEP peak-to-peak amplitude and AUC were also normalized to CMAP amplitude and AUC, for instance: (MEP_amplitude_/CMAP_amplitude_)*100%.

### Statistical analysis

To test whether group means of echogenicity and muscle thickness at baseline were different from normal values, a one sample t-test (test value: μ = 0) was performed. Baseline differences in echogenicity and muscle thickness between the paretic and non-paretic side were tested using paired samples t-tests. Generalized estimated equation modeling (GEE), with *time* (T0-T4) and *side* (paretic and non-paretic) as the independent variables, was used to assess changes over the follow-up period in the dependent variables: muscle echogenicity, muscle thickness, CMAP amplitude and AUC and (normalized) MEP amplitude and AUC. Since we expected that assessments obtained shortly after each other (e.g. T1 and T2) would be correlated stronger than assessments with a longer time interval (e.g. T2 and T3), we selected an autoregressive structure as the working correlation structure of the GEE model. In the case of a significant interaction of *side* by *time*, additional GEE analyses of *time* effects were performed for the paretic and non-paretic side, separately. These additional analyses of *time* per body *side* were corrected for multiple comparisons using Bonferroni correction. To assess changes in walking activity and capacity (steps per day and comfortable walking speed) Wilcoxon signed-rank tests were performed. We used SPSS (SPSS 15.0, SPSS Inc., Chicago, Illinois) for all statistical analyses. The level of significance was set at *p* < 0.05.

## Results

The characteristics of the included participants are presented in Table 1. One participant died before the first follow-up measurement, the cause of death being unrelated to the study; data of this participant was removed from the analysis. In one participant the FES system failed after 26 weeks. Since sufficient follow-up data were obtained, this participant was included in the final analysis. Hence, data of a total of 12 participants were used for final analysis. After 1 year of FES use the number of steps per day (6248 ± 3019, *p* = 0.59) and comfortable walking speed (1.02 ± 0.2 m/s, *p* = 0.09) were not significantly different from baseline values (5794 ± 2671 and 0.97 ± 0.2, respectively).

**Table 1:** Baseline group characteristics

N	13
Age; mean yrs. (SD)	56.1 (10.2)
Sex; male/female	10/3
Affected side; left/right	8/5
Type of stroke; ischaemic/ haemorrhagic	9/4
Years after stroke; mean yrs. (SD)	5.2 (4.5)
Body Mass Index; mean kg/m^2^(SD)	26.7 (3.4)
Fugl-Meyer Assessment – leg score (0–100%); median score (range)	71 (53–85)
Motricity Index – leg score (0–100%); median score (range)	72 (42–91)
Walking speed; mean m/s (SD)	0.97 (0.2)
Step count; mean steps/day (SD)	5794 (2671)

### Quantitative muscle ultrasound

#### Tibialis anterior

**Fig. 3:**
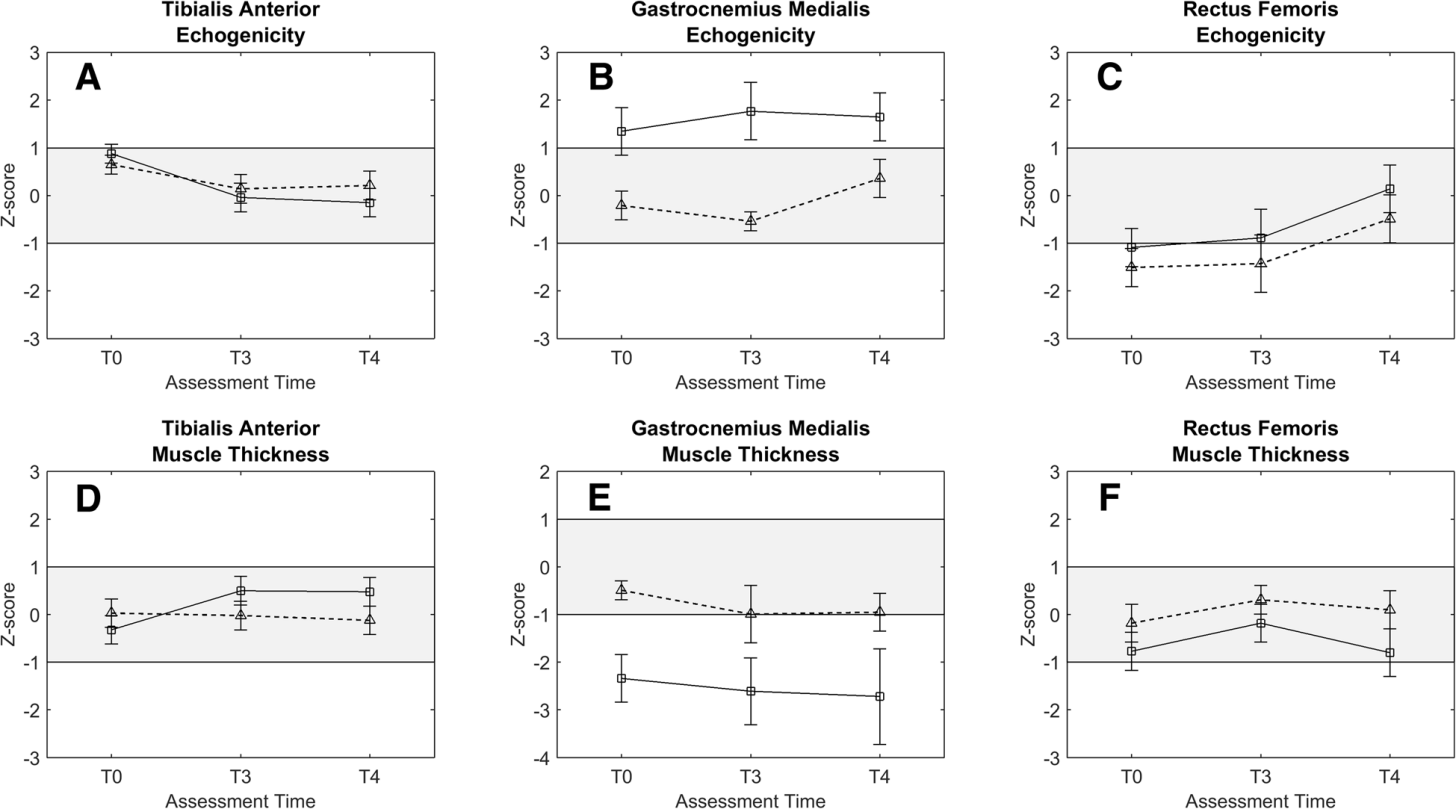
**a**-**f** Mean estimated z-scores ± standard errors for echogenicity and muscle thickness of the tibialis anterior (**a**, **d**), medial gastrocnemius (**b**, **e**), and rectus femoris (**c**, **f**) muscles over time: paretic side (circles, solid lines) and non-paretic side (triangles, dashed lines). Positive z-scores for echogenicity are considered indicative of poor muscle quality, whereas negative z-scores for muscle thickness indicate muscle atrophy. Grey bands represent the area within 1.0 SD of the reference values.Tables with estimated means and standard errors can be found in Additional file 2

At baseline, the mean echogenicity of the tibialis anterior muscle was significantly higher on both the paretic and non-paretic side (z = 0.88, *p* = 0.001, and z = 0.65, *p* = 0.008, respectively), meaning that the muscles looked more white and hence more structurally abnormal on the screen compared to reference values. Baseline echogenicity was not found to be significantly different between the paretic and non-paretic side. Over the follow-up period we found a decrease in tibialis anterior echogenicity which was most profound on the paretic side, as reflected by a significant interaction effect of *tim*e by *side (p* < 0.001*)*. Echogenicity z-scores on the paretic side were significantly different from baseline at T3 (*p* < 0.001) and T4 (*p* < 0.001) and decreased on average by 1.03 SD, indicating that muscle architecture improved over time. Remarkably, the echogenicity of the paretic side became lower than that of the non-paretic side at T4 (see Fig. 3a). We also found a significantly decreased echogenicity on the non-paretic side at T4 compared to T0, however, this finding was driven by imputation of two missing values at T4 by the GEE model. Additional parametric t-tests did not reveal a significant difference in echogenicity between baseline and T4 on the non-paretic side (*p* = 0.180).

At baseline, no significant differences compared to reference values *or* between the paretic and non-paretic side were found for muscle thickness of the tibialis anterior. Muscle thickness of the tibialis anterior on the paretic side increased over the follow-up period, being significantly different from baseline at T3 (*p* < 0.001) and T4 (*p* = 0.001; average increase of 0.80 SD, see Fig. 3b), whereas the muscle thickness on the non-paretic side did not (interaction *time* by *side, p* < 0.001).

#### Medial head of gastrocnemius

At baseline, the mean echogenicity of the medial head of the gastrocnemius muscle was significantly higher on the paretic side compared to reference values (*p* = 0.019) and compared to the non-paretic side (*p* = 0.007). The mean echogenicity did not change over time for either the paretic or non-paretic side. Overall, mean z-scores for echogenicity of the paretic and non-paretic side were 1.59 and − 0.13, respectively (see Fig. 3c).

Similar to the echogenicity pattern of the gastrocnemius, the paretic side muscle thickness at baseline was significantly smaller than reference values (*p* = 0.002) and compared to the non-paretic side (*p* = 0.006). Over the follow-up period muscle thickness remained significantly different between the paretic and non-paretic side (mean muscle thickness z-scores − 2.56 and − 0.81, respectively, *p* < 0.001), indicating that both sides showed reduced muscle thickness at a group level. Similar to the echogenicity, no significant changes in muscle thickness were found over time during FES use (see Fig. 3d).

#### Rectus femoris

At baseline, the mean echogenicity of the rectus femoris muscle was significantly lower than reference values on both the paretic side and non-paretic side (z = − 1.09, *p* = 0.014, and z = − 1.51, *p* = 0.002, respectively). Baseline echogenicity was not significantly different between the paretic and non-paretic side. During the follow-up period we found a significant change in echogenicity for both the paretic and non-paretic side at 52 weeks (T4) compared to T0 (*p* = 0.021). On average, echogenicity z-scores increased by 1.24 and 1.01 for the paretic and non-paretic side, respectively. Thus, after 1 year of FES use, mean echogenicity values for both the paretic and non-paretic side returned within one SD of the reference values (see Fig. 3e).

At baseline, mean rectus femoris muscle thickness on the paretic and non-paretic side were not significantly different from reference values. Paretic side muscle thickness was significantly smaller compared to the non-paretic side (*p* = 0.009), which was maintained over the follow-up period (*p* = 0.001). On average, paretic and non-paretic z-scores were − 0.58 and 0.08, respectively, indicating that only the paretic side showed reduced muscle thickness. No significant changes in rectus femoris thickness on either side could be detected over time (see Fig. 3f).

### Electrophysiologic assessment

#### Compound motor action potentials

**Fig. 4:**
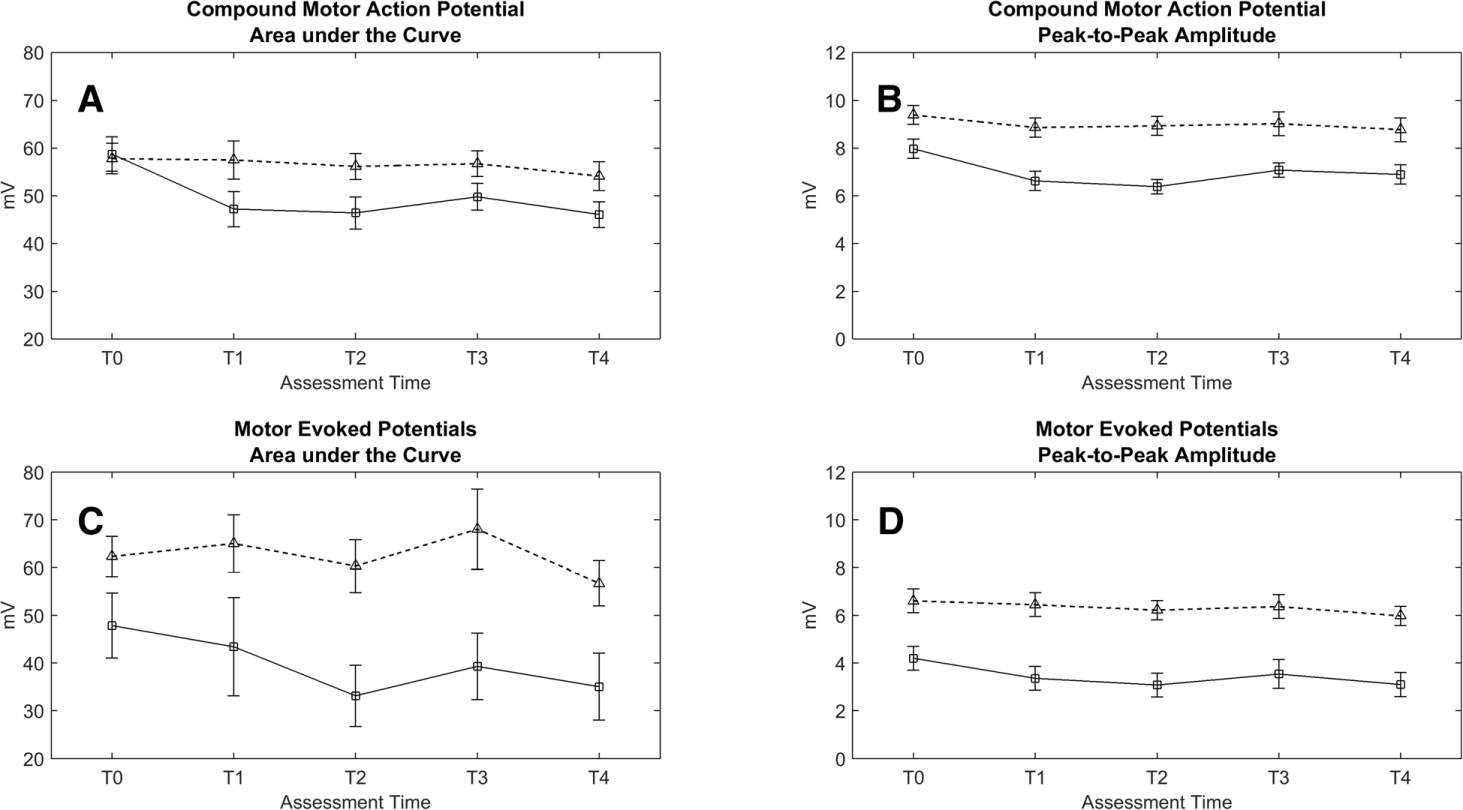
**a**-**d** Mean estimated area under the curve and peak-to-peak amplitudes ± standard errors for compound motor action potentials (**a**, **b**) and motor evoked potentials (**c**, **d**) obtained from tibialis anterior muscle over time: paretic side (circles, solid lines) and non-paretic side (triangles, dashed lines). Tables with estimated means and standard errors can be found in Additional file 2

The CMAP AUC and amplitudes of the tibialis anterior muscle were generally lower on the paretic side compared to the non-paretic side (*p* < 0.001). Following FES implantation, differences in CMAP AUC between the paretic and non-paretic side became larger (interaction *time* by *side p* < 0.002). This significant interaction of *time* by *side* was driven by a significant reduction in CMAP AUC on the paretic side (see Fig. 4a). It decreased from 58.7mVms (T0) to 46.0mVms (T4), and was found to be significantly different from baseline values during the entire follow-up period (*p* < 0.004). Similarly, an interaction of *time* by *side* was found for CMAP amplitudes (*p* < 0.005). Again, this interaction was driven by significant changes on the paretic side (see Fig. 4b). CMAP amplitudes on the paretic side decreased from 8.0 mV (T0) to 6.4 mV (T2, *p* < 0.001).

Subsequent analysis of the CMAP AUC and amplitudes showed no further changes from T1 or T2 to T4, indicating that the bilateral loss of CMAP amplitude/AUC after the surgical intervention was not fully reversed during the 1-year follow-up period.

#### Motor evoked potentials

MEP AUC and amplitudes of the tibialis anterior muscle were generally lower on the paretic side compared to the non-paretic side (*p* < 0.036). Similar to the CMAP AUC and amplitudes, the differences in MEP AUC between the paretic and non-paretic side became larger during the follow-up period, as was reflected by a significant interaction of *time* by *side* (*p* = 0.005). Also for MEP AUC, this significant interaction of *time* by *side* was due to a decrease on the paretic side, which was significantly different from baseline at T2 (*p* < 0.001). Between baseline and T2, mean MEP AUC decreased from 47.9mVms to 33.1mVms on the paretic side (see Fig. 4c).

MEP amplitudes on the paretic and non-paretic side decreased from 4.2 to 3.1 mV and from 6.6 to 6.0 mV, respectively. Although the average decrease in MEP amplitudes on the paretic side was nearly twofold the change on the non-paretic side,

no significant interaction of *time* by *side* was found (*p* = 0.119 at T2, see Fig. 4d). In general, MEP amplitudes were significantly different from baseline during the entire follow-up, T1-T4 (*p* < 0.046).

To assess whether changes over time in MEPs were due to changes in CMAPs we tested the effects of *time* and *side* on normalized MEPs. In contrast to MEP AUC, no significant interaction of *time* by *side* was found. Overall, normalized MEP AUC was significantly reduced, only at T2 (*p* < 0.001). With normalization of MEP amplitudes part of the *time* effects for MEP amplitudes, reported above, disappeared. With normalized MEP amplitudes significant differences in time were found only at T2 (*p* < 0.001) and T4 (*p* = 0.038).

## Discussion

Our study confirms that structural changes in skeletal muscle after a supratentorial stroke, reported here and elsewhere [9, 41–45], appear to be reversible with long-term FES use. Echogenicity of the tibialis anterior muscle decreased during the follow-up on the paretic side, which was accompanied by an increase in muscle size. Because of the chronic condition of the participants, it is unlikely that these changes are attributable to spontaneous recovery.

### Quantitative muscle ultrasound

In agreement with our hypothesis, the year-long use of an implanted peroneal FES stimulator successfully reversed the maladaptive changes to muscle structure in the tibialis anterior muscle. Previous studies have shown that FES can be successful in stopping or counteracting muscle atrophy, but this was done in other groups of patients (e.g., spinal cord injury) *and* through a resistance training program specifically aimed at muscle mass gain using surface-based FES stimulation [46–48]. The reduction in echogenicity on the paretic side of 1.03 SD comes down to a change in absolute echogenicity of approximately 20% [38], which is well over the 10% variation that could be expected between measurements [31]. Reduced echogenicity in the tibialis anterior muscle may have (partly) been caused by an increase in muscle mass [49], something we observed on the paretic side. However, the changes in echogenicity on the paretic side exceeded the changes in muscle thickness, which makes it unlikely that the observed decrease in echogenicity would be merely due to activity-induced muscle hypertrophy.

Since the implanted FES stimulator acts on the ankle dorsiflexor muscles, we hypothesized that no significant changes would occur in the medial gastrocnemius or rectus femoris muscle during 1 year of FES use. Although this was true for the medial head of the gastrocnemius, we did find changes in rectus femoris echogenicity during the follow-up period. Unexpectedly, and unlike the other muscles, the average echogenicity of the rectus femoris muscle was bilaterally lower than the reference values at baseline. This finding contrasts with the recent work by Akazawa et al. who reported increased echogenicity in the paretic rectus femoris muscles of sedentary and active people with stroke [45]. However, in the study by Akazawa et al., echogenicity was not corrected for age, sex, height or weight, as we did in the current study. As a result, direct comparison of the two studies is not possible. We have no explanation for the relatively low echogenicity of both rectus femoris muscles as observed in our study, however, our results indicate that this echogenicity was normalized after one-year of FES use. The normalization of echogenicity might point at a changed activity of the rectus femoris muscles as a result of FES use, at least on the paretic side, but this post-hoc explanation needs further investigation.

### Neurophysiologic assessment

Our results indicate that the CMAPs from the tibialis anterior muscle of the stimulated leg decreased after implantation of the FES system, a finding which we did not expect. The marked decline of the CMAP amplitudes on the paretic side (i.e. a loss of about 20%) could theoretically result from a reduction of functionally active motor units (e.g. due to nerve damage upon implantation) or from a lower excitability of motor unit components (i.e. axons, neuromuscular junctions and/or muscle fibers). Given the invasive character of the intervention, it is possible that the common peroneal nerve might have been damaged during surgery. However, if this would have been the case, we would have expected problems stimulating the tibialis anterior muscles *and* we would have expected signs of denervation of this muscle (i.e., increase in echogenicity and decrease in muscle size), which we did not find. We therefore believe that it is most likely that the excitability of the common peroneal nerve changed following implantation and, particularly, following activation of the FES stimulator. Indeed, axonal excitability can change under various conditions including overuse and fatigue [50, 51], and rapidly fatiguing muscles are a well-known problem in the application of FES [52]. Although participants were instructed to build up their daily FES use slowly and gradually, it might be that the paretic neuromuscular complex needed more time or was unable to adjust to the suddenly increased activation. Signs of fatigue were commonly reported by the participants, especially during the first weeks of follow-up. Additionally, it might be that the nature of FES provides a very different stimulation context for the motor axons than physiologic activation does, with concomitant changes in axonal resting and firing thresholds that may lead to a decrease in single-stimulus CMAP parameters.

In a number of studies, the use of FES has been shown to induce plastic changes on a cortical level [13–16]. Like in our study, Everaert et al. used MEPs to assess the effects of FES on cortical plasticity. In contrast to their work, we did not find signs of cortical plasticity after a year-long FES use. Instead, in our study, MEP amplitudes from the tibialis anterior muscle were even slightly reduced during the follow-up period. However, direct comparison of the results derived from both studies should be done with caution, given an important difference in measurement protocol. Where we used a fixed position of the magnetic stimulator for eliciting MEPs throughout the follow-up period, Everaert et al. aimed for the localization of ‘hot spots’ to achieve the best MEP response.

### Study limitations and recommendations

We assessed changes in three muscles in a relatively small and specific group of chronic stroke patients. The participants in our sample showed reduced ambulatory capacity, but were relatively active compared to the stroke community at large. The group in our study walked on average about 5800 steps per day, which is well above the number of steps reported in other studies [53]. To assure reliable imaging of muscles with muscle ultrasound, stroke survivors who were morbidly obese were excluded from the study. Since people with stroke are often obese and inactive, our results may therefore not be representative for the entire group of stroke survivors. However, our data show that even in active people with stroke structural changes in muscle architecture can be reversed, in our case by applying peroneal FES. Although we compared muscle characteristics to height-, weight-, sex- and age-corrected reference values, it is still uncertain whether the changes obtained in our study are clinically relevant. Future studies should aim at identifying cut-off criteria for relevant changes in both echogenicity and muscle thickness in this study population. In addition, other factors might influence muscle ultrasound characteristics in people with stroke, such as their hydration status that can change with the use of diuretics. The effects of such variables should be investigated in future research. Finally, this study focused on reversing the structural changes to muscle and nerves in people with chronic stroke (> 6 months post onset). It might be interesting to investigate whether FES could also be used to prevent such structural changes in the first months after a stroke.

## Conclusion

We have shown that the structural changes to muscles following supratentorial stroke are reversible with implanted peroneal FES and that these findings are not restricted to the stimulated ankle dorsiflexor muscles alone. We could not identify improvement of lower motor neuron functioning or cortical plasticity. The findings in this study add to the evidence that peroneal FES may have added value over the use of an ankle-foot orthosis in people with unilateral drop foot after stroke.

## Additional files

Additional file 1: Detailed description of the surgical procedure and system activation. (DOCX 17 kb)

Additional file 2: Tables with estimated means and standard errors on which the figures in the main manuscript were based. (DOCX 20 kb)

## Data Availability

The datasets used and/or analysed during the current study are available from the corresponding author on reasonable request.

## Abbreviations

AUC: Area under the curve
CMAPs: Compound motor action potentials
EMG: Electromyography
FES: Functional electrical stimulation
GEE: Generalized estimated equations
LMN: Lower motor neuron
MEPs: Motor evoked potentials
M-wave: Motor-wave
QMUS: Quantitative muscle ultrasound
ROI: Region of interest
UMN: Upper motor neuron
