# Supplementary material for: Long-term use of implanted peroneal functional electrical stimulation for stroke-affected gait: the effects on muscle and motor nerve
Source: J Neuroeng Rehabil. 2019 Jul 10;16:86. doi: 10.1186/s12984-019-0556-2 (PMC6621964; doi:10.1186/s12984-019-0556-2)
Supplement: Supplementary file 1 — Detailed description of the surgical procedure and system activation. (DOCX 17 kb) [file 12984_2019_556_MOESM1_ESM.docx]

#### Supplement A: Surgical procedure and system activation

#### ActiGait® implanted FES system

The ActiGait® system is an implantable 4-channel peroneal nerve stimulator (figure 2). The implant consists of an electrode cuff at the distal end and a stimulator body at the proximal end connected by a lead wire. The electrode cuff has 4 separate electrodes, which are selectively controlled by the stimulator body, allowing differential activation of nerve fibers to the tibialis anterior, peroneus longus/brevis, and toe extensor muscles. The system is operated through external parts: a heel switch (placed under the heel and attached to the shoe or a special sock) and a control unit, worn at the pelvis, which is connected to an antenna on the skin directly over the stimulator body. The control unit enables users to switch the stimulation on and off and to make adjustments in stimulation intensity. The heel switch wirelessly communicates with the control unit to provide information for onset and offset of stimulation .

#### Surgical procedure and system activation

ActiGait® implantation was performed by a trained neurosurgeon under general anaesthesia at the Radboud university medical center in Nijmegen. The full procedure of ActiGait® implantation has previously been described in detail (Burridge et al., 2007; Ernst et al., 2013, Schiemanck et al 2014). Optimal location for placement of the implant and surgical approach was determined by the surgeon pre-operatively based on an MRI scan of the paretic leg. Through a skin incision, the electrode cuff was placed around the common peroneal nerve distally to the branch of the lateral sural nerve and behind the distal tendon of the biceps femoris muscle. The stimulator body was implanted through a separate skin incision and attached to the superficial fascia approximately 10 cm distal to the greater trochanter. The lead wire was tunneled and placed in the subcutaneous fat layer avoiding longitudinal stress. Directly upon placement of the implant, the system was tested peroperatively to make sure that foot dorsiflexion and eversion were achieved through electrical stimulation. After closing the surgical wounds, X-ray images of the implant were taken with the knee in a flexed and extended position to check implant placement and stability. Participants were admitted to the neurosurgery ward for one or two days to ensure adequate post-operative care and guidance. In order for the wounds to heal and the implants to ‘settle down’, system activation was postponed until 3 weeks after surgery. An integrity check of the common peroneal nerve was performed before implantation as well as before first activation using electromyography (EMG): motor and sensory nerve conduction velocities of the superficial and deep peroneal nerve branches were tested and needle EMG of tibialis anterior, extensor digitorum brevis, and peroneal longus muscles was performed. Activation of the ActiGait® system was performed only if the postoperative EMG was comparable to the preoperative measurements. System activation was performed by a trained physiotherapist and the primary researcher (FB). Optimal individual system settings were based on a professional gait observation as well as on the subjective experience of the participant, to ensure optimal foot dorsiflexion during the swing phase, heel contact during initial contact, and controlled plantarflexion during the loading response without causing discomfort. Use of the Actigait® system was then increased stepwise from 15-60 minutes per day in the first week to at least 6 hours per day after three weeks. After this three-week familiarization period, the participants were allowed to use their FES system progressively as they saw fit.
