# Supplementary material for: Long-term use of implanted peroneal functional electrical stimulation for stroke-affected gait: the effects on muscle and motor nerve
Source: J Neuroeng Rehabil. 2019 Jul 10;16:86. doi: 10.1186/s12984-019-0556-2 (PMC6621964; doi:10.1186/s12984-019-0556-2)
Supplement: Supplementary file 2 — Tables with estimated means and standard errors on which the figures in the main manuscript were based. (DOCX 20 kb) [file 12984_2019_556_MOESM2_ESM.docx]

#### Supplement B: Tables with estimated means

1. Table 2: Mean estimated echogenicity z-scores (standard errors).

|  | Tibialis Anterior | | Gastrocnemius | | Rectus Femoris | |
| --- | --- | --- | --- | --- | --- | --- |
|  | Paretic | Non-paretic | Paretic | Non-paretic | Paretic | Non-paretic |
| T0 | 0.88 (0.2) | 0.65 (0.2) | 1.35 (0.5) | -0.21 (0.3) | -1.09 (0.4) | -1.51 (0.4) |
| T3 | -0.04 (0.3) | 0.14 (0.3) | 1.77 (0.6) | -0.54 (0.2) | -0.89 (0.6) | -1.43 (0.6) |
| T4 | -0.15 (0.3) | 0.21 (0.3) | 1.65 (0.5) | 0.36 (0.4) | 0.14 (0.5) | -0.49 (0.5) |

1. Table 3: Mean estimated muscle thickness z-scores (standard errors).

|  | Tibialis Anterior | | Gastrocnemius | | Rectus Femoris | |
| --- | --- | --- | --- | --- | --- | --- |
|  | Paretic | Non-paretic | Paretic | Non-paretic | Paretic | Non-paretic |
| T0 | -0.32 (0.3) | 0.03 (0.3) | -2.34 (0.5) | -0.49 (0.2) | -0.77 (0.4) | -0.18 (0.4) |
| T3 | 0.50 (0.3) | -0.02 (0.3) | -2.61 (0.7) | -0.99 (0.6) | -0.18 (0.4) | 0.31 (0.3) |
| T4 | 0.48 (0.3) | -0.12 (0.3) | -2.72 (1.0) | -0.95 (0.4) | -0.80 (0.5) | 0.10 (0.4) |

Table 4: Mean estimated CMAP amplitude and area under the curve (standard errors).

|  | CMAPs:  peak-to-peak amplitude (mV) | | CMAPs:  area under the curve (mVms) | |
| --- | --- | --- | --- | --- |
|  | Paretic | Non-paretic | Paretic | Non-paretic |
| T0 | 8.0 (0.4) | 9.4 (0.4) | 58.7 (3.6) | 57.8 (3.2) |
| T1 | 6.6 (0.4) | 8.9 (0.4) | 47.2 (3.7) | 57.5 (4.0) |
| T2 | 6.4 (0.3) | 8.9 (0.4) | 46.4 (3.4) | 56.1 (2.7) |
| T3 | 7.1 (0.3) | 9.0 (0.5) | 49.8 (2.8) | 56.7 (2.7) |
| T4 | 6.9 (0.4) | 8.8 (0.5) | 46.0 (2.7) | 54.1 (3.0) |

CMAPs= Compound motor action potentials from tibialis anterior muscle

Table 5: Mean estimated (normalized) MEP amplitude and area under the curve (standard errors).

|  | MEPs:  peak-to-peak amplitude (mV) | | MEPs:  area under the curve (mVms) | | MEPs:  normalized peak-to-peak amplitude | | MEPs:  normalized area under the curve | |
| --- | --- | --- | --- | --- | --- | --- | --- | --- |
|  | Paretic | Non-paretic | Paretic | Non-paretic | Paretic | Non-paretic | Paretic | Non-paretic |
| T0 | 4.2 (0.5) | 6.6 (0.5) | 47.9 (6.8) | 62.3 (4.2) | 52.7 (6.2) | 70.3 (4.4) | 80.5 (8.6) | 108.5 (5.5) |
| T1 | 3.4 (0.5) | 6.5 (0.5) | 43.4 (10.3) | 65.0 (6.0) | 49.6 (6.5) | 72.7 (4.5) | 86.7 (17.3) | 110.6 (8.0) |
| T2 | 3.1 (0.5) | 6.2 (0.4) | 33.1 (6.4) | 60.3 (5.5) | 45.9 (6.2) | 70.2 (4.8) | 66.9 (9.2) | 107.2 (5.1) |
| T3 | 3.5 (0.6) | 6.4 (0.5) | 39.3 (7.0) | 68.0 (8.4) | 49.1 (7.7) | 71.7 (5.0) | 74.2 (9.5) | 118.7 (12.5) |
| T4 | 3.1 (0.5) | 6.0 (0.4) | 35.1 (7.0) | 56.7 (4.7) | 45.4 (7.4) | 68.9 (4.1) | 76.0 (15.1) | 105.3 (7.3) |

MEPs= Motor evoked potentials from tibialis anterior muscl
